# Water for Thought: Is There a Role for Aquaporin Channels in Delirium?

**DOI:** 10.3389/fpsyt.2014.00057

**Published:** 2014-05-26

**Authors:** Adonis Sfera, Carolina Osorio

**Affiliations:** ^1^Psychiatry, Patton State Hospital, Patton, CA, USA; ^2^Kaiser Permanente, Fontana, CA, USA

**Keywords:** delirium, aquaporins, neuroinflammation, acetylcholine, aquaporin blockers

C. G. Jung believed that water was a symbol for the unconscious mind, the background from where our conscious thoughts emerge, and also the sea where they melt into the dream-like state of primary processes (1).

Water comprises about 80% of the brain volume and water homeostasis is inextricably coupled to the CNS function. In this regard, neuroscience describes an inverse relationship between the intensity of the neuropil function and the amount of water it contains. For example, during high neuronal activity (such as information processing), water is transported away from the neuropil, shrinking the extracellular matrix (ECM). Conversely, during times of lower brain activity (such as during sleep), water is shifted back to the neuropil, expanding the ECM (2, 3).

The movement of water in and out of the neuropil occurs with the help of the glymphatic system via special molecular pumps, aquaporin water channels (AQP 4) located in astrocyte end-feet. Water circulation is enabled by the exchange between the cerebrospinal fluid (CSF) and interstitial fluid (ISF). The pressure gradient for this exchange is probably provided by pericytes’ contraction and arterial pulsations along with the suction, pump-like action of AQP 4 channels (4–6). This movement of water in and out of the neuropil enables both, clearance of molecular waste and volume transmission (VT) of chemical signals (7). Conversely, delayed water movement (glymphatic stasis) may predispose to the accumulation of misfolded proteins (4) and ultimately to neuroinflammation (8).

The relationship between water and delirium is complex. Both, brain edema and dehydration may predispose to delirium (9). Up-regulation of AQP 4 water channels seems to occur in both situations. In fact, a biphasic up-regulation was described in edema build-up and the resolution phase (10). Interestingly, AQP 4 receptors seem to be the common denominator between the neuropil water movement and neuroinflammation (10). Moreover, animal studies demonstrated that peripheral dehydration triggers central up-regulation of AQP 4 receptors (11–13). This in turn causes swelling and priming of astrocytes and microglia, predisposing to neuroinflammation (14).

According to a recent study, two key factors, systemic inflammation and central cholinergic impairment must interact in order to produce delirium (15).

The goal of this article is not to discuss VT or the relationship between AQP 4 channels and inflammation since extensive literature exists on these subjects. Instead, we attempt to answer three questions:

Can an inefficient glymphatic clearance lead to impairment of central cholinergic transmission?

Does glymphatic stasis contribute to microglia and astrocytes’ priming, the precursor of neuroinflammation?

Do aquaporin blockers have a place in delirium?

We hypothesize that failure of glymphatic clearance leads to impairment of acetylcholine volume transmission (AChVT), contributing to impaired arousal, attention, memory, and sleep as seen in delirium.

We hypothesize further, that glymphatic failure is pro-inflammatory in nature, leading to up-regulation of AQP 4 channels, which in turn trigger astrocyte swelling and gliosis with the end result being microglial and astrocytic priming.

The above phenomena may reconcile two theories of delirium: central cholinergic deficit and neuroinflammation.

## Acetylcholine and Glymphatic Stasis

Central cholinergic deficit is the best established neurotransmitter dysfunction in delirium and its role was known for a long time, however, impaired AChVT was not given much thought in the literature in spite of evidence demonstrating that 86–93% of cholinergic boutons in the CNS do not make synaptic contact, but release acetylcholine (ACh) directly into the ISF (16).

It is generally accepted that chemical communication between neurons can occur by fast, point to point transmission at the synapse, or by non-synaptic interactions in which neurotransmitters are released from axon terminals (without a conventional synaptic contact) directly into the ISF. In this later case, neurotransmitters travel through the ISF by convection and influence the activity of other neurons through stimulation of extrasynaptically located receptors (17).

Failure of glymphatic clearance may lead to accumulation of molecular debris in the ISF, which in turn may impair the “go-with-the-flow” of AChVT. Since VT represents a large proportion of ACh signaling, it was hypothesized that it may support the sustained and widespread neural functions such as cognition, attention, awareness, and sleep (18, 19). Since these functions are affected in delirium and since low plasma and CSF levels of ACh have been consistently described in delirious patients (20), it is possible to assume that AChVT may play a central role in the pathophysiology of delirium.

The discovery of the glymphatic clearance helps put AChVT in perspective vis-à-vis neurodegeneration, sleep, and possibly delirium. The link between AQP 4 and cholinergic neurotransmission was demonstrated by animal studies. For example, AQP 4 and chlorine receptors (ClCN3) up-regulation was documented in studies of transgenic mice with Ach deficit (21).

Another interesting animal study documented co-localization of cholinergic muscarinic receptors (mAChRs) and aquaporin-4 (AQP4) water channels on astrocytic membrane (22). Microglia is known to express nicotinic receptors on its membrane. It is also known that activation of these receptors *in vitro* attenuates pro-inflammatory responses (23). Since microglia also expresses AQP 4 receptors (24), it is possible, that just like in astrocytes, glymphatic stasis leads to a deficit of AChVT and up-regulation of AQP 4 channels on microglial surface. AQP 4 up-regulation (which is pro-inflammatory) may constitute the link between cholinergic deficit and neuroinflammation.

On the other hand, the lack of efficacy of rivastigmine in delirium (25) may not invalidate the cholinergic deficit theory. Instead, it may demonstrate the difficulty in correcting ACh function while AQP 4 receptors are up-regulated. It would be interesting to study the efficacy of rivastigmine used concomitantly with an AQP 4 blocker in delirium.

## Neuroinflammation and Glymphatic Stasis

The brain lacks a lymphatic circulation or macrophages. For this reason, neuroinflammation is immunologically different from peripheral inflammation. In this respect “priming,” which occurs in the CNS prior to neuroinflammation, can be fathomed as being similar to sensitization. Priming was described in both microglia and astrocytes, cells that also express AQP 4 water channels on their surface. In preclinical animal models, it was demonstrated that in response to the accumulation of abnormally folded proteins in the ISF, astrocytes and microglia react by adopting an activated state, and by releasing molecules that drive their own proliferation. When “primed” these cells are susceptible to a secondary inflammatory stimulus that may arise from surgery or other systemic inflammatory process (8). Interestingly, normal aging was also demonstrated to be a stimulus for microglial “priming” (26).

The process of priming may explain both, the increased incidence and the sudden onset of delirium in medically and surgically ill patients (20).

The accumulation of molecular waste in the ISF also affects neuropil hydration, leading to impairment in neuronal excitability and survival. Genetic studies demonstrate the existence of hydration-sensitive genes in the brain, such as clathrin, that influence neuroexcitability, trigger glial swelling, and result in neuropathology (27).

In a 2013 study, our group created a rat model of ischemia of the nucleus basalis of Meynert and hippocampus. Ischemic changes consisted of swelling of astrocytes, pericyte dysfunction, detachment of astrocytic end-feet from the capillaries with release of glial fibrillary acidic protein (GFAP). AQP4 receptors were not directly assessed, but astrocyte edema (visualized by CD3 staining of GFAP antibodies) provided indirect evidence of AQP4 up-regulation (28).

Up-regulation of AQP 4 receptors was described in the pathophysiology of delirium due to liver failure (29). Cultured astrocytes treated with ammonia have been shown to undergo cell swelling with increased expression of AQP4 receptors (30).

AQP4 water receptors up-regulation was demonstrated in traumatic brain injury (TBI) (31), ischemia, epilepsy, multiple sclerosis, HIV encephalitis, and progressive multifocal leukoencephalopathy (PML) (32).

Studies of Alzheimer’s disease demonstrated enhanced expression of astrocytic AQP4 receptors compared to age-related controls (33). These changes may be caused by the process of aging, especially since it was documented that in senescence astrocytes up-regulate both AQP 4 and Kir 4.1 potassium receptors (34). Moreover, studies in older mice demonstrate up-regulation of these receptors, perhaps in order to maintain homeostasis, integrative ability, and adaptation (34). It is interesting that in diabetes mellitus, type 2, the brain also presents with astrocytic swelling, probably caused by up-regulation of AQP 4. This response was shown to be more pronounced around zones of infarction and it causes delay in vascular repair in the post-stroke period. One study showed that metformin prevented astrocyte swelling and facilitated re-vascularization (35). Neuromyelitis optica (NMO) is an inflammatory demyelinating disease that typically affects optic nerves and spinal cord. Autoantibodies against AQP4 are implied in its etiology of NMO (36).

Conversely, decreased expression of AQP4 receptors on astrocyte membrane was found to be neuroprotective (37). For example, in a mouse model of ischemia using AQP4 null mice, intracranial pressure elevation, blood–brain barrier disruption, inflammation, brain edema, and neuronal apoptosis were shown to be reduced in comparison to AQP4 positive mice (38).

Interestingly, melatonin, and melatonin agonist ramelteon were recently found beneficial in delirium (39). The idea of using melatonin in delirium is not new, given that sleep fragmentation and circadian rhythm changes were described previously. The novelty concerns the mechanism of action of melatonin with its ability to block protein kinase C, which inhibits AQP 4 expression (40). In addition, melatonin was shown to be anti-inflammatory, to inhibit nitric oxide synthase and dopamine release (41).

Additional studies are needed to clarify whether neuropsychiatric conditions associated with up regulation of AQP 4 receptors are more likely to predispose to delirium as opposed to situations in which AQP 4 channels are not up-regulated.

## Do Aquaporin Blockers have a Place in Delirium?

Studies of ischemia and inflammation in AQP 4-null mice demonstrated decreased astrocytic swelling, and improved overall outcomes and survival (14). With these findings in mind, it was suggested that pharmacological modulation of AQP4 expression may provide a new addition to the medical armamentarium of disorders such as brain edema, glaucoma, tumor growth, CHF, and obesity in which water and solute transport are involved (42). Since AQP 4 up-regulation, astrocytic swelling and microglia/astrocyte priming may occur in delirium as a result of glymphatic stasis, would it be reasonable to expect that AQP 4 blockers might be beneficial in delirium?

A newly discovered arylsulfonamide (Aq B013) is an antagonist of AQP 1 and AQP 4. This is a pharmacologic agent that offers a translational promise in the treatment of conditions manifested by up-regulation of aquaporins (43). Could it also be beneficial in delirium?

In an animal study, piroxicam was demonstrated to be a potent AQP4 regulator, rendering neuroprotection in focal cerebral ischemia (44). In NMO, an attempt was made at blocking pathogenic NMO–IgG binding to its target, AQP4 by recombinant monoclonal anti-AQP4 antibodies (45).

## Conclusion

Impairment of cognition, attention, awareness, and sleep are encountered both, in delirium and Ach deficit. Because the implementation of these functions requires sustained and widespread neuronal activity, it has been proposed that they may be promoted better by volume, rather than synaptic transmission of Ach, which is best suited for fast and selective signals.

Glymphatic stasis has two possible consequences that may be of interest for delirium: impairment of AchVT and priming of microglia and astrocytes. Both of these phenomena are associated with up-regulation of AQP 4 receptors, which may be the common denominator between neuroinflammation and cholinergic deficit. Aquaporin blockers, which were found to be useful in cerebral ischemia and stroke, may also be beneficial in delirium alone or in addition to cholinesterase inhibitors.

## Conflict of Interest Statement

The authors declare that the research was conducted in the absence of any commercial or financial relationships that could be construed as a potential conflict of interest.
